# The Impact of Socio-Economic Status on Self-Rated Health: Study of 29 Countries Using European Social Surveys (2002–2008)

**DOI:** 10.3390/ijerph10030747

**Published:** 2013-02-25

**Authors:** Javier Alvarez-Galvez, Maria Luisa Rodero-Cosano, Emma Motrico, Jose A. Salinas-Perez, Carlos Garcia-Alonso, Luis Salvador-Carulla

**Affiliations:** 1 Department of Social Work and Social Policy, Universidad Loyola Andalucia, Seville 41014, Spain; E-Mails: mlrodero@uloyola.es (M.L.R.-C.); emotrico@uloyola.es (E.M.); jsalinas@uloyola.es (J.A.S.-P.); 2 Department of Management and Quantitative Methods, Universidad Loyola Andalucia, Cordoba 14004, Spain; E-Mail: cgarcia@uloyola.es; 3 Centre for Disability Research and Policy, Faculty of Health Sciences, University of Sydney, Sydney 2141, Australia; E-Mail: luis.salvador-carulla@sydney.edu.au

**Keywords:** European countries, self-rated health (SRH), socio-economic status (SES), health inequalities, education

## Abstract

Studies show that the association between socio-economic status (SES) and self-rated health (SRH) varies in different countries, however there are not many country-comparisons that examine this relationship over time. The objective of the present study is to determine the effect of three SES measures on SRH in 29 countries according to findings in European Social Surveys (2002–2008), in order to study how socio-economic inequalities can vary our subjective state of health. In line with previous studies, income inequalities seem to be greater not only in Anglo-Saxon and Scandinavian countries, but especially in Eastern European countries. The impact of education is greater in Southern countries, and this effect is similar in Eastern and Scandinavian countries, although occupational status does not produce significant differences in southern countries. This study shows the general relevance of socio-educational factors on SRH. Individual economic conditions are obviously a basic factor contributing to a good state of health, but education could be even more relevant to preserve it. In this sense, policies should not only aim at reducing income inequalities, but should also further the education of people who are in risk of social exclusion.

## 1. Introduction

Social and economic circumstances have an impact on our physical and mental health. Evidence has shown that the higher the socio-economic status (SES), the lower the prevalence and/or incidence of health problems, illness, disease and death [1,2]. The relationship between SES and self-rated health (SRH) varies in strength among countries [3,4]. Income inequalities vary from one country to another and these economic determinants could produce differences in individuals’ states of health [5,6,7,8]. 

However, there are not many country-comparisons that examine the impact of SES on SRH over time. Most studies have been limited to comparing a small set of countries and usually analyzing one year in a transversal design that cannot describe variations due to diverse effects over time [9]. Moreover, the results of these studies show unexpected findings. Although it could be supposed that Scandinavian countries with higher levels of public social spending present higher levels of SRH, empirical studies reliably demonstrate that income-related health inequalities are even smaller in Central European countries than in Scandinavia. Even though the latter region is characterized by having a relatively generous package of social policies, the promotion of social equality and universal welfare provision (including health care), the lowest degree of inequality is actually found in Central European countries. Meanwhile, it is the Southern and Eastern countries that are found to have the greatest health inequalities, due to their fragmented and underdeveloped welfare system [10,11,12,13,14]. 

On the other hand, while many studies are focused on economic determinants of health in Europe, there is still a lack of studies trying to explain these inequalities using social factors [15,16,17]. In fact, the measurement of income inequalities does not include other social dimensions that are relevant to understanding differences in subjective health. A wide range of SES factors contribute to explaining differences in SRH, such as social class [18,19], differences in the job market [20] or the educational systems [15,16]. Hanibuchi and colleagues have compared the relationship between different measures of SES (income, education, occupational status and class identification) and SRH in four East Asian countries (China, Japan, South Korea and Taiwan) [21]. The study evidences a statistically significant association between SES and SRH, and their variations in the four countries selected. In this study, income shows a stronger relationship to SRH than occupation status or education, while class identification exhibited the strongest association. But how do these relationships work in European countries? And has this pattern been changing in the first decade of the XXI century?

In order to respond to these questions, the main objective of the present study is to compare the effect of different measures of SES on SRH in 29 countries according to European Social Surveys (ESS) during the period 2002–2008. That is, the purpose of this study is to examine how socio-economic inequalities can vary our subjective state of health over time, and thus shed light on future trends. 

## 2. Method

### 2.1. Data and Variables

In order to explain the differences in net SRH between European countries, an analysis has been performed on a cumulative dataset comprising the four waves of the European Social Survey (the ESS cross-sectional design includes the following years: 2002, 2004, 2006 and 2008) [19]. This dataset has a sample size of 185,154 units at individual level. The 29 countries included in our analysis are Austria, Belgium, Bulgaria, Cyprus, Czech Republic, Denmark, Estonia, Finland, France, Germany, Greece, Hungary, Ireland, Israel, Italy, Luxembourg, Netherlands, Norway, Poland, Portugal, Russian Federation, Slovenia, Slovakia, Spain, Sweden, Switzerland, Turkey, Ukraine and the United Kingdom. Although Israel cannot be classified as European, it has been included since this country is included in the European Social Survey. The target population covered those 15 years or older who are residents within private households, regardless of nationality or citizenship, language or legal status. A more detailed description of the ESS is provided elsewhere [22]. 

The dependent variable is the self-rated general state of health (SRH) which was included in the ESS questionnaire in question C7 (“How is your health in general? Would you say it is...”), with a 5 likert-point answer (1 “Very bad”; 2 “Bad”; 3 “Fair”; 4 “Good”; 5 “Very good”). 

According to Hanibuchi *et al.* [21], different explanatory variables (*i.e.*, SES measures) were included in the analysis. There were three variables: income, education and occupational status (see descriptive statistics in Table 1). Subjective social class was not considered since this variable is absent in ESS. In ESS data, household total net income is reported in 12 banded categories that are standardized for 29 countries (for more information on this variable visit the following link: http://ess.nsd.uib.no/), although this variable is treated as continuous by taking the midpoint of each banded category [23]. Education is measured as years of completed full-time education. Occupational status is measured using the International Socio-Economic Index of Occupational Status (ISEI) [24]. The ISEI is computed using a causal model that, while controlling for age, takes into account occupational status, education and income, and obtains scores for each occupation by the optimal scaling of the occupational unit group in the ISCO88 classification [25]. This variable did not present multicollinearity with the other variables and has no effect on the overall test of the model or on model predictions.

**Table 1:** Descriptive statistics for variables in the model.

	Obs.	Mean	Std. Dev.	Min.	Max.
*SRH*	184,718	3.759	0.933	1	5
Income	129,344	14,217.69	24,390.57	0	180,000
Education	182,739	11.855	4.116	0	56
Occ. status	160,280	42.083	16.611	16	90

Once the statistical relevance at the bivariate level had been determined between the three predictors and the dependent variable, the ordinal regression model was carried out. These variables had been standardized previously in order to enable statistical comparisons.

### 2.2. Statistical Methodology

The SRH mean values for the four years have been mapped for each country in a Geographic Information System to allow better visualization of the results per country. Map intervals have been delimited by natural breaks, where the cut-offs are defined by seeking natural groups in the data distribution. The GIS searches the lower points in the histogram of SRH means which delimit similar values and maximize differences between classes.

Given that the response variable is an ordinal category with five possible values, an Ordered Logistic Regression (OLR) model was performed to estimate the effect of the explanatory variables [26,27]. Brant’s test [28] for proportional odds was assessed to provide evidence that the parallel assumption had not been violated. In the proportional odds model, which is a direct generalization of the binary logistic regression model, the odds ratios between each pair of levels is assumed to be the same, regardless of which two adjacent levels are chosen. Thus, the odds ratio from this model for our five level ordinal regression is actually a weighted average of the four individual odds ratios, as we increase from one level to the next. The four individual odds ratios are assumed to be the same by the model and, thus, the odds ratio in this model is fairly robust.

According to Greene [25], the model is built around a latent regression as follows:

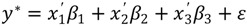
(1)

Being *x1*: Income, *x2*: Education, *x3*: Occupational Status. As usual, y* is unobserved. What we do observe is:

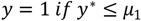


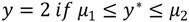


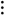


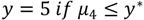


Then the ordered logit technique will use the observations on *y*, which are a form of censored data on y*, to fit the parameter vector. We assume that *ε* is normally distributed across observations, giving the following probabilities:

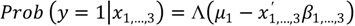


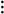


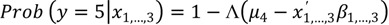


For all the probabilities to be positive, we must have:

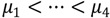


Once the models were computed, the goodness of fit test was appropriate and statistically significant. The obtained Pseudo *R*^2^ ranged between 0.01 and 0.10. 

## 3. Results

**Figure 1:**
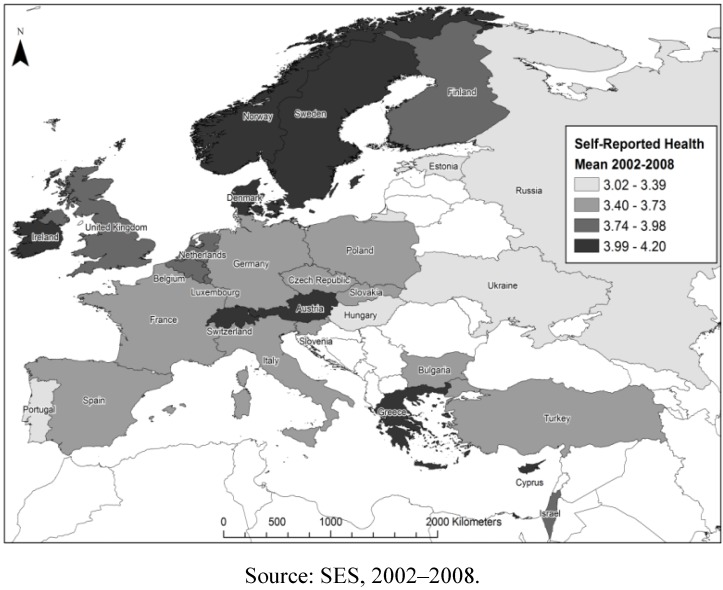
Mean values of self-rated health in 29 countries based on European Social Surveys (2002–2008). Self-rated health intervals by natural breaks.

Figure 1 shows the mean differences in SRH for the 29 countries in the ESS sample for the period 2002–2008. Differences between years for the dependent variable were not shown on the map since these were minimal. All countries have a generalized positive perception of their health. In comparison with other European countries, the Northern ones report a better general state of health, especially Scandinavian and Anglo-Saxon countries (Ireland, United Kingdom, Denmark, Finland, Norway, and Sweden). As an exception, Switzerland, Austria, Greece and Cyprus also report a similar positive auto-evaluation. On the other hand, people living in Eastern countries, such as Estonia, Ukraine, Hungary or Russia present the opposite trend when reporting their subjective state of health. Among Mediterranean countries, Portugal has a poor rating in the SRH scale. Central and Southern countries position themselves in the middle of this rating.

Table S1 summarizes the results of ordered logistic regression analysis (see Appendix). This table shows the standardized coefficients for the three SES measures. Variations in SRH among countries are evident in the results of the models that were computed. However, some regularities can be found when comparing different European regions. The models demonstrated the association between SES and SRH throughout the period 2002–2008, especially for Southern and Western European countries where the results of the goodness of fit test were better. That is, socio-economic status explains a greater proportion of SRH variance in these countries, which is an evidence of more social and economic inequalities in the less developed regions of Europe. 

**Figure 2:**
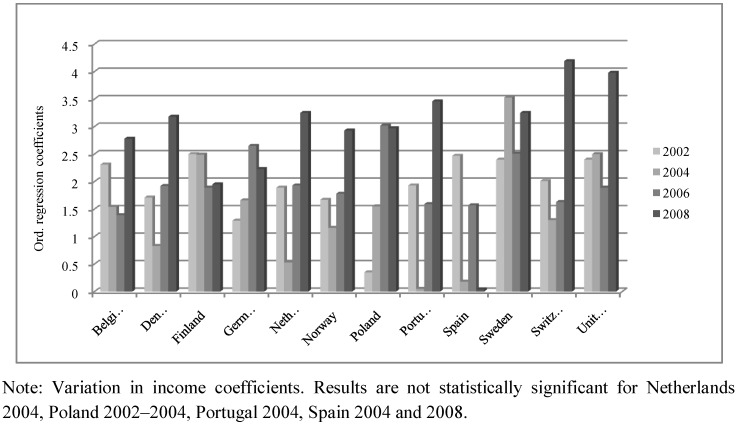
Variation for the impact of income on self-rated health.

**Figure 3:**
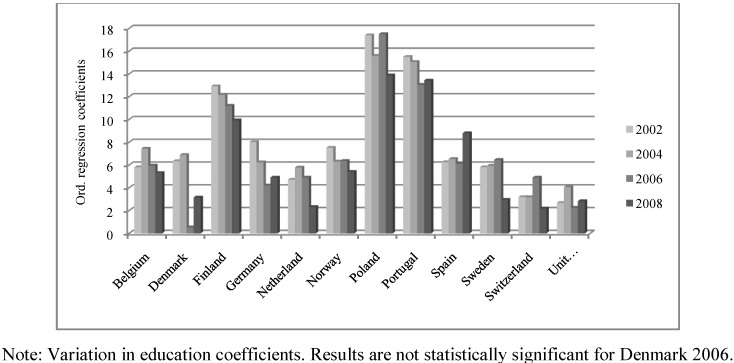
Variation for the impact of education on self-rated health.

**Figure 4:**
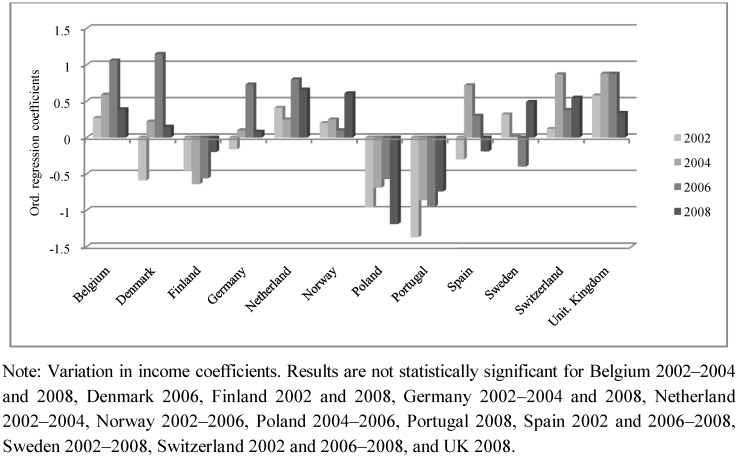
Variation for the impact of occ. status on self-rated health.

Additionally, in order to facilitate the interpretation of the results, we have included three figures (Figure 2, Figure 3, Figure 4) that describe differences among the coefficients of the three independent SRH variables (income, education and occupational status) in twelve countries. These countries have been selected because they have the complete year sequence and are representative of different regions of Europe.

Figure 2 shows the effect of income on SRH. Among Scandinavian countries, we can observe a generalized increase in the impact of income on SRH in the period 2002–2008 with the exception of Finland. Despite this difference the coefficients are relatively similar to those of Norway, Sweden and Denmark. The United Kingdom presents a similar pattern to Scandinavian ones for the positive effect of income on SRH, while Ireland does not reproduce this increasing trend. In Central European countries, there is a generalized increase in the effect of income on people’s subjective health status throughout the period 2002–2008. That is, income-related health inequalities have increased in Northern and in Central European countries during last few years (see as an example the case of Austria, Belgium, Switzerland, Germany, France and the Netherlands). In general, Southern countries do not present clear differences when comparing the impact of income on SRH, and this effect is not always statistically significant (e.g., Spain, a country with a public health system). Finally, income differences are comparatively greater in Eastern countries than in Northern ones, but it is not possible to identify a clear trend (see Table S1).

The impact of education is described in Figure 3. On the one hand, the higher association of education with SRH as compared to income is quite relevant. If we look at the ranges of coefficients for the two variables, the relevance of education is seen to even triplicate that of income (income range: 0.5 to 4.0; education range: 0.2 to 16.0). On the other hand, there is a steady decrease in education-related differences these years, especially in Scandinavian and Central European countries. That is, the impact of education on individuals’ general health has fallen moderately during this period. In the UK, as an exception, the impact of education remains relatively constant in the period 2002–2008 and it is even lower than the effect of income. For that reason, education has a mild impact in the UK, whilst it is not statistically significant in Ireland. In Central European countries, the positive relationship of education with SRH is still higher than that of income but, on the other hand, this relationship does not follow a stable pattern. In general terms, we can say that the effect of education progressively decreases during this period in Belgium, Germany, France, Netherlands and Switzerland. There are, however, some exceptions (e.g., Austria, where this effect is growing).

The main characteristic of Southern/Mediterranean and Eastern countries is the comparatively higher relevance of education when explaining the differences in SRH. In line with the general pattern described in Figure 3, these differences have declined, for example, in Portugal and Poland (and Hungary, Greece, Slovenia and Turkey in Table S1). As an exception, education-related inequalities seem to be greater in Spain during these years, while for other countries it is difficult to find a clear trend concerning the effect of education because of the lack of data (Bulgaria, Cyprus, Estonia and Ukraine).

Finally, Figure 4 shows the impact of occupational status. The effect of this predictor is not clear since the relationship of this variable with SRH persistently varies over time, and does not show a regular trend or stable statistical significance compared to the other SES measures. Denmark, for example, presents a negative association between occupational status and SRH for the year 2002 (Table S1), whilst this effect is reversed in 2006. The positive association between occupational status and SRH is much clearer than in Northern countries, but the effect of this variable is still weaker than that produced by income and education. On the other hand, since the statistical significance of occupational status is restricted to isolated years, it is difficult to describe a pattern that can shed light on the relationship of this variable with individuals’ subjective states of health. Finally, it is interesting to observe the negative association of occupational status and SRH in Southern and Eastern countries.

## 4. Discussion

This study provides a comparison between three socioeconomic determinants of health (income, education and occupational status), and describes the evolution in the relationship of these measures with SRH in European countries for the period 2002–2008. In line with previous studies, these results point out the relevance of socio-economic factors in explaining the differences in individuals’ states of health [9,10,11,12]. The self-perceived general health of people living in Scandinavian and Anglo-Saxon countries was observed to be less affected by SES determinants in comparison to Southern and East European countries [11]. 

Despite the differences between countries, this study indicates the relevance of education in preserving a good general state of health during the period 2002–2008 [8]. Even though other SES measures such as income or occupational status are important to explain health-related inequalities during the evaluation period, educational status is the main contributor to explain the differences in SRH, especially in countries characterized by having less flexible economies and a fragmented system of welfare provision (*i.e*., Southern/Mediterranean and Eastern/Post-Communist) [29,30]. For example, among these countries it is interesting to consider the case of Spain, which is characterized by having a general public health system. This fact explains that, in comparison with other countries with basic levels of provision and public social spending (*i.e*., Anglo-Saxon), income-related inequalities are considerably reduced and some cases are not statistically significant in Spain, which is characterized by universal access to the public health system. Thus, income-related inequalities are greater in the United Kingdom, a country characterized by minimal levels of provision and modest social transfers, although this trend is also reproduced in central European and Scandinavian countries. This tendency increased during the period 2002–2008 and it is expected to get even stronger in the last years of the financial global crisis and during the gradual liberalization of European economies [31,32]. On the other hand, the effect of education is progressively decreasing in many different European countries. This pattern has probably been associated with the effect of social expenditure in public education in Europe, that is, the general increasing of educated population in this region [33].

Occupational status loses in statistical significance and this effect is weaker that that produced by income and education. Therefore it is difficult to follow a clear pattern that can describe and explain the relationship of this variable with individuals’ subjective states of health. As mentioned, the effect of occupational status is not equally correlated with SRH in all European countries. In fact, this correlation remains clearly positive among Anglo-Saxon and Central/European countries, but negative among Southern and Eastern countries. This negative association evidences how, in these fragmented and less developed welfare regimes, income guarantees are linked to work positions and a higher variation in income maintenance schemes [31,34]. In other words, having a high occupational position in the labor markets of Southern and Eastern countries does not guarantee a better state of health, maybe because salaries are not high enough to favor a more positive perception of general subjective health.

Unlike present budget cuts, the new context of the global financial crisis should demand more education and new distributive policies to avoid not only a decline in individual socio-economic status, but also deterioration in the general state of health of the European population. These findings evidence that, in the current crisis context, there is a need to: (1) continue allocating public social spending on education to reduce health differences; (2) continue reducing both health inequalities generated by income differences and also (3) those related to occupational status and labor markets. 

Even though the privatization of less efficient parts of European public systems may be needed to increase the flexibility that new global markets demand, current and future policy-making must take into account the complex interaction that is maintained between people’s socio-economic conditions and their subjective general health. Income, education and occupation-related inequalities cannot determine the individuals’ state of health in objective terms, but the lack of these factors is likely to deteriorate our physical and mental condition. Furthermore, it should be highlighted that education, having the strongest association with SRH, seems to mediate the association of income and occupation inequalities. That is, education is a previous step to practice a healthy lifestyle, even for wealthier and upper class people. In this sense, the educational component must be considered a core factor in explaining these health-related inequalities and in reducing them, thereby obliging policy-makers to consider the relevance of this social determinant.

We face three main limitations in interpreting our results. First, there is a problem of causation in the actual relationship between the independent variables and the dependent variable when using cross**-**sectional design for each wave of ESS data. Second, our research focuses on individual determinants of subjective general health, though in the future it might be relevant to study the impact of contextual determinants of health inequalities and therefore to include other variables as controls (these incorporations may increase the small R^2^ values). Third, the period of analysis comprises a series of years of relative stability in the economic market. Thus, in the future it will be important to analyze the effects of the financial and debt crisis and, in some specific cases, there are unreliable estimations due to a lack of data (e.g., Russian Federation, Ukraine, Bulgaria or Slovakia). Taking these limitations into account, it might be relevant to incorporate new ESS waves to extend the period of study, to improve the statistical representativeness of different countries (specially Eastern ones) and therefore the consistency of the results over time; in turn, the effect of the global financial crisis on Europeans’ state of health could be specifically analyzed.

## 5. Conclusions

This work analyzes the current evolution in the relationship of three SES measures and SRH in 29 European countries for the period 2002–2008. As we have shown, in this period individual economic conditions are indispensable factors to enjoy a good state of health, but education seems to still be a more important determinant of our subjective well-being, even despite the decrease described in its association with SRH in the period studied. That is the reason why new European policies should not only aim to reduce economic inequalities, but also to increase the education of people, which can actively contribute to maintaining a good state of health. 

Of course, taking into consideration the current context of the global financial crisis, we must be cautious since the relationship between income and SRH has been increasing in recent years. This study shows that education plays a crucial role in improving our health, but the data that we use do not capture the worsening of the economic situation in the last years, in particular in the South of Europe. In fact, it is relevant to notice how the relationship of income with SRH increases slightly during these years and possibly this association will grow in a context of socioeconomic polarization. Despite the fact that the European Union has tried to reduce health inequalities, these differences still persist. Income-related health inequalities have not disappeared for the most part, and have actually increased in the period 2002–2008. In this sense, specific attention still has to be paid to the socioeconomic factors that determine people’s state of health, especially in those mechanisms that reproduce the poor health of excluded groups and/or individuals living in risk of social exclusion in Europe. 

## Appendix

**Table S1:** Results of ordered logistic regression analysis (ESS, 2002–2008). Standardized coefficients.

	Year 2002	Year 2004	Year 2006	Year 2008
*Austria*				
Income	1.90 (0.61) ^***^	1.64 (0.60) ^***^	2.30 (0.53) ^***^	NA
Education	5.42 (1.32) ^***^	7.51 (1.56) ^***^	7.06 (1.36) ^***^	NA
Occ. status	0.82 (0.39) ^**^	−0.13 (0.42)	0.65 (0.35) ^*^	NA
R^2^	0.02	0.02	0.03	NA
*Belgium*				
Income	2.31 (0.62) ^***^	1.54 (0.50) ^***^	1.39 (0.50) ^***^	2.78 (0.54) ^***^
Education	5.81 (1.27) ^***^	7.45 (1.12) ^***^	5.96 (1.17) ^***^	5.30 (1.24) ^***^
Occ. status	0.27 (0.35)	0.59 (0.36)	1.06 (0.33) ^***^	0.39 (0.34)
R^2^	0.02	0.03	0.03	0.03
*Bulgaria*				
Income	NA	NA	62.88 (8.25) ^***^	NA
Education	NA	NA	10.81 (1.74) ^***^	NA
Occ. status	NA	NA	−1.10 (0.46) ^**^	NA
R^2^	NA	NA	0.06	NA
*Cyprus*				
Income	NA	NA	3.34 ^***^ (1.17)	NA
Education	NA	NA	15.38 ^***^ (1.80)	NA
Occ. status	NA	NA	−0.89 (0.57)	NA
R^2^	NA	NA	0.09	NA
*Czech Republic*				
Income	6.69 (1.67) ^***^	5.01 (0.89) ^***^	NA	9.47 (1.70) ^***^
Education	9.95 (1.83) ^***^	12.02 (1.81) ^***^	NA	8.45 (2.00) ^***^
Occ. status	−0.22 (0.50)	0.42 (0.39)	NA	0.16 (0.41)
R^2^	0.03	0.03	NA	0.03
*Denmark*				
Income	1.71 (0.50) ^***^	0.83 (0.43) ^*^	1.92 (0.45) ^***^	3.18 (0.56) ^***^
Education	6.35 (1.26) ^***^	6.89 (1.25) ^***^	0.54 (0.84)	3.16 (0.89) ^***^
Occ. status	−0.59 (0.35) ^*^	0.22 (0.34)	1.15 (0.32) ^***^	0.15 (0.32)
R^2^	0.03	0.02	0.02	0.02
*Estonia*				
Income	NA	NA	NA	5.16 (0.57) ^***^
Education	NA	NA	NA	11.85 (1.32) ^***^
Occ. status	NA	NA	NA	−0.49 (0.32)
R^2^	NA	NA	NA	0.07
*Finland*				
Income	2.50 (0.58) ^***^	2.49 (0.51) ^***^	1.89 (0.46) ^***^	1.95 (0.50) ^***^
Education	12.93 (1.03) ^***^	12.16 (1.01) ^***^	11.21 (0.97) ^***^	9.93 (0.92) ^***^
Occ. status	−0.45 (0.30)	−0.64 (0.29) ^**^	−0.56 (0.30) ^*^	−0.20 (0.27)
R^2^	0.06	0.06	0.06	0.05
*France*				
Income	NA	1.18 (0.44) ^***^	1.48 (0.55) ^***^	2.57 (0.51) ^***^
Education	NA	9.70 (1.03) ^***^	7.75 (0.94) ^***^	7.47 (0.96) ^***^
Occ. status	NA	−0.34 (0.32)	−0.30 (0.31)	−0.30 (0.30)
R^2^	0.03	0.03	0.03	0.03
*Germany*				
Income	1.29 (0.44) ^***^	1.66 (0.41) ^***^	2.65 (0.47) ^***^	2.23 (0.61) ^***^
Education	8.03 (1.08) ^***^	6.25 (1.12) ^***^	4.21 (1.04) ^***^	4.91 (1.05) ^***^
Occ. status	−0.16 (0.29)	0.10 (0.30)	0.73 (0.30) ^**^	0.08 (0.29)
R^2^	0.02	0.02	0.02	0.01
*Greece*				
Income	3.29 (0.81) ^***^	6.29 (1.08) ^***^	NA	1.43 (0.84) ^*^
Education	14.09 (1.08) ^***^	14.36 (1.09) ^***^	NA	13.90 (1.40) ^***^
Occ. status	−1.21 (0.41) ^***^	−1.50 (0.42) ^***^	NA	−0.74 (0.49)
R^2^	0.08	0.10	NA	0.06
*Hungary*				
Income	NA	12.25 (2.41) ^***^	NA	5.27 (0.86) ^***^
Education	NA	13.20 (1.67) ^***^	NA	6.62 (1.47) ^***^
Occ. status	NA	−0.63 (0.42)	NA	−0.09 (0.46)
R^2^	NA	0.05	NA	0.04
*Ireland*				
Income	NA	2.52 (0.42) ^***^	1.23 (0.40) ^***^	2.05 (0.70) ^***^
Education	NA	6.00 (1.19) ^***^	6.07 (1.35) ^***^	6.48 (1.11) ^***^
Occ. status	NA	0.30 (0.33)	0.15 (0.37)	0.20 (0.32)
R^2^	NA	0.04	0.02	0.03
*Israel*				
Income	3.60 (0.76) ^***^	NA	NA	0.11 (0.54)
Education	3.15 (1.09) ^***^	NA	NA	7.05 (1.24) ^***^
Occ. status	−0.15 (0.31)	NA	NA	−0.05 (0.31)
R^2^	0.01	NA	NA	0.01
*Italy*				
Income	3.40 (1.01) ^***^	1.66 (0.61) ^***^	NA	NA
Education	8.27 (1.77) ^***^	9.23 (1.41) ^***^	NA	NA
Occ. status	−0.25 (0.65)	−0.44 (0.49)	NA	NA
R^2^	0.05	0.04	NA	NA
*Luxemburg*				
Income	1.25 (0.60) ^**^	1.02 (0.53) ^*^	NA	NA
Education	4.53 (1.48) ^***^	5.99 (1.22) ^***^	NA	NA
Occ. status	0.83 (0.45) ^*^	0.31 (0.42)	NA	NA
R^2^	0.02	0.03	NA	NA
*Netherland*				
Income	1.89 (0.39) ^***^	0.53 (0.44)	1.93 (0.47) ^***^	3.25 (0.57) ^***^
Education	4.72 (0.88) ^***^	5.79 (1.06) ^***^	4.90 (0.91) ^***^	2.33 (0.96) ^**^
Occ. status	0.41 (0.29)	0.25 (0.33)	0.80 (0.33) ^**^	0.66 (0.35) ^*^
R^2^	0.02	0.02	0.03	0.03
*Norway*				
Income	1.67 (0.34) ^***^	1.16 (0.29) ^***^	1.78 (0.27) ^***^	2.93 (0.52) ^***^
Education	7.53 (1.11) ^***^	6.31 (1.07) ^***^	6.37 (1.01) ^***^	5.41 (1.05) ^***^
Occ. status	0.20 (0.31)	0.25 (0.31)	0.10 (0.31)	0.61 (0.32) ^*^
R^2^	0.03	0.02	0.03	0.03
*Poland*				
Income	0.35 (0.85)	1.55 (1.08)	3.02 (1.17) ^**^	2.97 (0.60) ^***^
Education	17.40 (1.33) ^***^	15.60 (1.50) ^***^	17.50 (1.52) ^***^	13.89 (1.49) ^***^
Occ. status	−0.96 (0.37) ^**^	−0.69 (0.42)	−0.57 (0.40)	−1.19 (0.40) ^***^
R^2^	0.06	0.05	0.07	0.06
*Portugal*				
Income	1.93 (0.86) ^**^	0.05 (0.69)	1.59 (0.66) ^**^	3.46 (1.47) ^**^
Education	15.51 (1.36) ^***^	15.06 (1.29) ^***^	13.06 (1.26) ^***^	13.43 (1.48) ^***^
Occ. status	−1.37 (0.48) ^***^	−0.86 (0.48) ^*^	−0.95 (0.50) ^*^	−0.74 (0.57)
R^2^	0.09	0.08	0.07	0.08
*Russian Federation*				
Income	NA	NA	31.13 (3.29) ^***^	4.84 (0.54) ^***^
Education	NA	NA	13.52 (1.24) ^***^	15.06 (1.24) ^***^
Occ. status	NA	NA	−0.56 (0.32) ^*^	−1.18 (0.30) ^***^
R^2^	NA	NA	0.08	0.08
*Slovenia*				
Income	7.82 (1.62) ^***^	7.56 (1.69) ^***^	8.28 (1.46) ^***^	5.23 (0.81) ^***^
Education	10.63 (1.60) ^***^	9.14 (1.80) ^***^	8.65 (1.56) ^***^	6.43 (1.42) ^***^
Occ. status	−0.61 (0.42)	0.03 (0.46)	0.58 (0.44)	−0.19 (0.40)
R^2^	0.05	0.05	0.06	0.05
*Slovakia*				
Income	NA	1.78 (1.11)	7.75 (1.56) ^***^	NA
Education	NA	7.55 (1.79) ^***^	9.11 (1.63) ^***^	NA
Occ. status	NA	−0.73 (0.42) ^*^	−0.02 (0.42)	NA
R^2^	NA	0.01	0.04	NA
*Spain*				
Income	2.47 (1.24) ^**^	0.18 (0.83)	1.57 (0.64) ^**^	0.04 (0.66)
Education	6.26 (1.08) ^***^	6.54 (1.08) ^***^	6.12 (0.96) ^***^	8.80 (0.90) ^***^
Occ. status	−0.30 (0.50)	0.72 (0.43) ^*^	0.30 (0.43)	−0.19 (0.37)
R^2^	0.03	0.04	0.04	0.04
*Sweden*				
Income	2.40 (0.63) ^***^	3.53 (0.57) ^***^	2.51 (0.49) ^***^	3.25 (0.52) ^***^
Education	5.82 (1.06) ^***^	5.94 (1.07) ^***^	6.46 (1.08) ^***^	2.96 (1.06) ^***^
Occ. status	0.32 (0.29)	0.01 (0.29)	−0.40 (0.30)	0.49 (0.30)
R^2^	0.02	0.03	0.03	0.02
*Switzerland*				
Income	2.01 (0.35) ^***^	1.30 (0.34) ^***^	1.63 (0.35) ^***^	4.19 (0.71) ^***^
Education	3.19 (1.11) ^***^	3.18 (1.10) ^***^	4.90 (1.21) ^***^	2.19 (1.19) ^*^
Occ. status	0.12 (0.33)	0.87 (0.31) ^***^	0.38 (0.37)	0.55 (0.38)
R^2^	0.02	0.02	0.03	0.03
*Turkey*				
Income	NA	−0.90 (1.21)	NA	2.02 (0.97) ^**^
Education	NA	7.25 (1.48) ^***^	NA	4.91 (1.47) ^***^
Occ. status	NA	−0.27 (0.54)	NA	−0.82 (0.48) ^*^
R^2^	NA	0.02	NA	0.01
*Ukraine*				
Income	NA	NA	NA	6.46 (0.83) ^***^
Education	NA	NA	NA	10.26 (1.28) ^***^
Occ. status	NA	NA	NA	−0.31 (0.32)
R^2^	NA	NA	NA	0.06
*United Kingdom*				
Income	2.40 (0.34) ^***^	2.50 (0.40) ^***^	1.89 (0.30) ^***^	3.98 (0.47) ^***^
Education	2.68 (1.07) ^**^	4.07 (1.31) ^***^	2.26 (0.85) ^***^	2.84 (0.92) ^***^
Occ. status	0.58 (0.28) ^**^	0.88 (0.31) ^***^	0.88 (0.26) ^***^	0.34 (0.26)
R^2^	0.03	0.03	0.02	0.03

Notes: **^***^**, **^**^**, and **^*^** indicate significance level at 1%, 5%, and 10%, respectively. Standard errors in brackets. NA: Information not available.
